# A Developmental Framework for Embodiment Research: The Next Step Toward Integrating Concepts and Methods

**DOI:** 10.3389/fnsys.2021.672740

**Published:** 2021-07-30

**Authors:** Vanessa Lux, Amy L. Non, Penny M. Pexman, Waltraud Stadler, Lilian A. E. Weber, Melanie Krüger

**Affiliations:** ^1^Department of Genetic Psychology, Faculty of Psychology, Ruhr-Universität Bochum, Bochum, Germany; ^2^Department of Anthropology, University of California, San Diego, La Jolla, CA, United States; ^3^Department of Psychology, University of Calgary, Calgary, AB, Canada; ^4^Chair of Human Movement Science, Department of Sports and Health Sciences, Technical University of Munich, Munich, Germany; ^5^Department of Psychiatry, Oxford Centre for Human Brain Activity, Warneford Hospital, Oxford, United Kingdom; ^6^Translational Neuromodeling Unit, Institute for Biomedical Engineering, University of Zurich and ETH Zurich, Zurich, Switzerland; ^7^Institute of Sports Science, Faculty of Humanities, Leibniz University Hannover, Hannover, Germany

**Keywords:** embodied experiences, agency approach, environmental approach, developmental systems theory, language acquisition, cognition, perception, interoception

## Abstract

Embodiment research is at a turning point. There is an increasing amount of data and studies investigating embodiment phenomena and their role in mental processing and functions from across a wide range of disciplines and theoretical schools within the life sciences. However, the integration of behavioral data with data from different biological levels is challenging for the involved research fields such as movement psychology, social and developmental neuroscience, computational psychosomatics, social and behavioral epigenetics, human-centered robotics, and many more. This highlights the need for an interdisciplinary framework of embodiment research. In addition, there is a growing need for a cross-disciplinary consensus on level-specific criteria of embodiment. We propose that a developmental perspective on embodiment is able to provide a framework for overcoming such pressing issues, providing analytical tools to link timescales and levels of embodiment specific to the function under study, uncovering the underlying developmental processes, clarifying level-specific embodiment criteria, and providing a matrix and platform to bridge disciplinary boundaries among the involved research fields.

## Introduction

Embodiment has become a key concept in human life sciences in recent years. Although generally understood as the sum of bodily preconditions of cognition, emotion, and behavior, a closer look unveils that conceptualizations of embodiment vary strongly among research areas and theoretical schools: from embodied simulation to embodied cognition, from biological embedding of experience to interoception and the embodied mind. Differences also include types of knowledge production (e.g., cognitive processing models vs. motor developmental pathways vs. computational simulations of neural activity patterns), data modalities (e.g., self-reports vs. medical diagnoses vs. movement trajectories vs. molecular epigenetic profiles), and the involved levels of analysis (e.g., psychological function vs. gene expression). Different research areas and theoretical schools also account for different timescales across which embodiment processes can be analyzed (e.g., life-time effects vs. acute activation). Importantly, some of the most pressing topics with the greatest potential to advance embodiment research cut across research disciplines and are currently significantly hampered by the lack of a common theoretical framework that bridges the different conceptualizations of embodiment.

The current situation calls for an interdisciplinary conceptualization of embodiment. Here, we propose to adopt a developmental perspective as an integrative cross-disciplinary framework of embodiment research serving three main purposes: A developmental perspective on embodiment *accounts for the different timescales* that underlie the processes of incorporation and expression of an agent's embodied experiences in the interaction with the environment. It also *relates different levels of embodiment to one another* as they develop over the lifespan, based on their biological and functional interconnectedness. Finally, it *clarifies disciplinary boundaries* and finds connection points by identifying transmission hubs between the levels involved in a particular embodiment process which, then, creates links for collaboration between participating disciplines. This is especially important for research areas which try to overcome disciplinary limits but are still bound to the methods and committed to research standards within their respective field. Thus, on the basis of those three purposes, the proposed framework does not aim at providing a new definition of embodiment but shall be understood as a platform and analytical tool to enable and support integrative cross-disciplinary embodiment research by clarifying critical aspects that currently hamper the advance in the field.

In the following, we will, first, characterize two main types of approaches to embodiment in the life sciences, agency and environmental approaches (see section Integrating Environmental and Agency Approaches to Embodiment) and discuss how they conceptualize developmental processes underlying embodiment phenomena (see section Developmental Processes Grounding Embodiment: Implicit and Underexplored). Due to the variety of embodiment research in the life sciences, these two sections do not cover all existing embodiment concepts. We focus on those fields of embodiment research and concepts of embodiment for which adapting an integrative developmental perspective would be most productive from our perspective. Second, we will present examples for the potential and the challenges of developmental embodiment research (see section Developmental Embodiment Research: Cross-Disciplinary Examples). Finally, we discuss our framework of developmental embodiment research in more detail (see section Toward an Integrative Framework for Developmental Embodiment Research).

## Integrating Environmental and Agency Approaches to Embodiment

Developmental embodiment research, as we propose it in this paper, builds on two distinct lines of current embodiment research in the life sciences, which rarely connect conceptually, or in empirical studies (see Box 1 and Table 1 for an introduction into key concepts, targeted embodiment phenomena and related key references): First, “*agency approaches*” that emphasize how bodily embeddedness, anatomic preconditions, and physiological as well as neurophysiological foundations momentarily enable movements, actions, and psychological functions. Second, “*environmental approaches*,” which describe how the physical, social, and cultural environment is incorporated, and affects the physical structure of the body or brain and subsequently its function. Agency approaches mainly focus on the acute activation and involvement of the body, including basal body functions, signals, resources, and conditions etc. with emphasis on the active interaction with the environment. Research originating from an agency approach investigates how embodied experiences are mobilized and how they influence interactions with the environment. Environmental approaches, in contrast, focus on the long-term impact of environmental signals on bodily preconditions of mental functions and mental health outcomes. Research coming from an environmental approach investigates how environmental (pre)conditions and events are incorporated into the body to become embodied experiences. With our developmental framework, we propose to integrate agency and environmental approaches by understanding the body as a reservoir of experiences, providing a sort of storage and ‘memory' of experiences and capabilities necessary for action and mental functions which are activated and mobilized in the specific moment of interaction with the environment.

Box 1 **Approaches to embodiment in the life sciences which contribute to the proposed developmental perspective.**

The rise of embodiment concepts in the life sciences started out in the 1980s, when linguists, cognitive psychologist, and robotic engineers turned to the study of motor and sensory processing, emphasizing that they shape the way our mind works. Neuroscience and philosophy of mind contributed to further advance this perspective (Varela et al., 1991; Gibbs, 2005; Shapiro, 2011). Subsequently, a range of different concepts developed by building up on the joint idea that our bodily preconditions are the foundation for the perception of ourselves (in the sense of consciousness and interoception) and the perception of the environment with which we interact, affecting a range of processes and phenomena related to e.g., cognition, emotion processing, social interaction, aesthetic perception, and mental health. We subsume these as agency approaches to embodiment (see Table 1).

While the lines along which different embodiment concepts should be distinguished are still highly debated (for embodied cognition, being the most prominent concept of an agency approach to embodiment, see e.g., Clark, 1999; Wilson, 2002; Overton et al., 2008; Kiverstein, 2012; Meteyard et al., 2012), they were picked up quickly in cognitive psychology and other related fields of research (see Table 1). Here, they provided a platform for the use of the newly established neurofunctional approach based on neuroimaging techniques (mainly fMRI) within the existing cognitive paradigm. In sports psychology and movement science, for example, the concepts of embodied cognition and embodied simulation bridged the gap between neurocognitive research and practical knowledge based on motor expertise, movement learning, as well as motor rehabilitation techniques. Further, the acknowledgment of the embeddedness of our mind within our most basal bodily functions via interoceptive pathways (see Craig, 2004, 2009a,b) and their influence on emotion processing, self-awareness, and time perception, strongly influenced research fields such as computational psychiatry and psychosomatics, and mental health research (Petzschner et al., 2017; Khalsa et al., 2018). Here, we already observe first steps to integrate agency approaches to embodiment with those studying processes of incorporation of experiences at several underlying biological levels (Petzschner et al., 2017).

This turn to the bodily preconditions of action, perception, and emotions met with a turn to the body in population health and epidemiological research (see Krieger, 2005; Hertzman, 2012; Rutter, 2012; Gluckman et al., 2016). The question was whether and how adverse or advantageous environmental conditions, experienced during sensitive periods in life (e.g., pre- or perinatal), become incorporated into the body and subsequently constitute divergent developmental pathways of physical and mental health. The concepts used to describe such long-term effects of environmental impacts, which we subsume as environmental approaches to embodiment (see Table 1), focus on different physiological levels of the body underlying mental functions, e.g., (psycho)physiological feedback mechanisms, hormone regulation, neural networks, brain anatomy, inflammatory processes, gene-environment interactions, as well as epigenetic mechanisms (e.g., Godfrey et al., 2007; Wadhwa et al., 2009; Danese et al., 2011; Rutter, 2012; Nelson, 2017; Bush et al., 2018; Aristizabal et al., 2019). We limit our discussion here to those studying the impact on mental health. While these concepts differ in their focus on the time-point and duration of the environmental impact, spanning transient to persistent effects, they all attribute a central role to developmental processes in the translation of the environmental impact to later mental health outcome. Thus, the classification of developmental pathways underlying diverse outcomes is a joint important goal in these concepts. It is also assumed necessary for translational research and future intervention (Rutter, 2016).

**Table 1:** Examples of agency and environmental approaches to embodiment: concepts, phenomena and fields of research.

**Agency approaches**		**Environmental approaches**	
**Concepts**			
Embodied cognition	Wilson, 2002; Leitan and Chaffey, 2014; Shapiro, 2014	Biological embedding of experiences	Danese et al., 2011; Rutter, 2012; Nelson, 2017; Bush et al., 2018; Aristizabal et al., 2019
Embodied simulation	Gallese and Goldman, 1998; Gallese, 2007, 2017, 2019	Developmental Origin of Health and Disease (DOHaD)	Barker, 1995, 1998; Hanson and Gluckman, 2008; Gluckman et al., 2016
Somatic marker hypothesis	Damasio, 1994, 1996	Environmental epigenetics	Weaver et al., 2004; Zhang and Meaney, 2009; Bollati and Baccarelli, 2010
Inference-control loop	Petzschner et al., 2017		
Phenomenological approaches to embodiment	MacLachlan, 2004; Gallagher, 2005; Thompson, 2007; Fuchs, 2008		
Bio-looping	Seligman et al., 2015; Kirmayer and Gómez-Carrillo, 2019		
**Phenomena**			
Abstract mental processing	Pfeifer and Bongard, 2006; Zdrazilova et al., 2018	Bullying	Mulder et al., 2020
Action perception	Buxbaum and Kalénine, 2010; Gredebäck and Falck-Ytter, 2015	Gender	Zhang et al., 2018
Aesthetic judgement	Kirsch et al., 2016; Gallese, 2017, 2019	Enriched environments	Cortes et al., 2019
Emotion contagion	Fawcett et al., 2016, 2017	Racial discrimination	Brody et al., 2016
Emotion perception	Adolphs, 2002; Vermeulen and Mermillod, 2010	Socio-economic status	Needham et al., 2015; McDade et al., 2019
Interoception	Craig, 2004, 2009b; Seth, 2013; Seth and Friston, 2016	Traumatic events	Ramo-Fernández et al., 2015; Kuan et al., 2017
Joint action	Sebanz et al., 2006; Vesper et al., 2010		
Joint attention	Moore et al., 1995; Eilan, 2005		
Language development	Rizzolatti and Arbib, 1998; Fuchs, 2016b; Inkster et al., 2016; Sidhu and Pexman, 2016		
Mental health	Herbert and Pollatos, 2012; Petzschner et al., 2017; Khalsa et al., 2018		
Motor imagery	Lotze and Halsband, 2006; Filimon et al., 2007; Munzert et al., 2009		
Social perception and judgement	IJzerman and Semin, 2010; Kang et al., 2011; Meier et al., 2012		
**Fields of research**			
Cognitive psychology Computational psychiatry Human movement science Robotics Social psychology Sports psychology		Anthropology Epidemiology Genetic psychology Social psychiatry	

Other attempts to classify embodiment approaches often distinguish a third group of phenomenological approaches (Overton, 2008), or further differentiate along methods (experiential/objective), epistemological perspective (1st, 2nd, and 3rd third person perspective), and object of research (cognitive structure/practice) (Hornecker et al., 2017). We subsume phenomenological approaches under the group of agency approaches, as these are also primarily concerned with the momentary preconditions of consciousness, action, or perception, although from a first (or second) person perspective.

Integrating agency and environmental approaches to embodiment clearly broadens the notion of embodiment compared to its use in discipline-specific research areas (e.g., in embodied cognition research, see Wilson, 2002). In addition to different neural networks involved, e.g., in decision making, abstract word recognition, and movement execution, embodiment processes also encompass the physical constitution of the body (the anatomic structure of the limbs, homeostatic feedback processes, hormonal balance, basic sensory organization, and function, etc.), the neural networks involved in the regulation of these processes, and the molecular underpinnings of these regulation processes and network constitutions. This broader notion of embodiment further includes the focus on the anatomic structure of the body used in robotics (see Pfeifer and Bongard, 2006), the notion of an extended mind to our immediate environment and, most importantly, intersubjectivity emphasized in phenomenological accounts (see Fuchs, 2017), as well as the biological embedding of experiences across the lifespan (see Rutter, 2012). Still, not all body processes and conditions are of interest for developmental embodiment research, but only those which participate in the incorporation, shaping, and expression of embodiment experiences, in the context of e.g., a particular mental function or mental health outcome under study. Developmental embodiment research, as we propose it here, provides a matrix and platform through which different approaches of embodiment research can collaborate in the interdisciplinary study of a particular mental function or mental health outcome.

## Developmental Processes Grounding Embodiment: Implicit and Underexplored

Although developmental theories are one of the historical pillars of embodiment research (Overton and Lerner, 2012), the question of how embodied functions develop often comes second in empirical studies. Some authors even question that a developmental perspective contributes at all to elucidate the underlying mechanisms of embodiment phenomena (e.g., Körner et al., 2015). In our view, both the agency approaches and the environmental approaches to embodiment imply developmental processes and would therefore profit from an explicit developmental perspective.

*Agency approaches*, on the one hand, implicitly acknowledge lifespan developmental changes in e.g., cognitive and motor functioning within their theoretical framework, but the specific impact on the embodiment phenomena under study is not empirically investigated. Referencing Gibson's ecological theory (Gibson, 1986//2014) or Piaget's stages of cognitive development (Inhelder and Piaget, 1958; Piaget, 1977), for example, agency approaches emphasize that the mind develops through an individual's interactions with the material world around it. Consequently, (inter-) individual differences in e.g., perception, imagination, language processing, or aesthetic judgement, as well as in neuronal activity measured in the sensorimotor cortex are understood as having evolved from these interactions. The activation of interoceptive and motor processing networks during these higher cognitive tasks then works as an indicator for the degree of bodily groundedness of a function.

Studies of developmental changes of embodiment phenomena based on the agency approach are rare, with exceptions often focusing on action perception and speech development during infancy and early childhood (Wellsby and Pexman, 2014; Gredebäck and Falck-Ytter, 2015; Fuchs, 2016b; Gottwald et al., 2016; Inkster et al., 2016; Gredebäck, 2018; Loucks and Sommerville, 2018). However, a number of studies examines changes of embodiment phenomena on a much shorter timescale due to e.g., training effects, therapeutic interventions, or short-term manipulation and impairment of motor capabilities (e.g., Koch et al., 2008; Marasco et al., 2011; Meugnot et al., 2014; Kuehn et al., 2018). It is likely that the mechanisms underlying short-term plastic changes of embodiment processes overlap with those underlying the longer-term developmental changes (Wenger et al., 2017). To fully bridge these timescales, however, agency approaches need to be integrated with environmental approaches.

*Environmental approaches* to embodiment, on the other hand, explicitly examine the developmental outcome related to the environmental impact under study. The goal is to trace the underlying biological processes, which lead to the observed associations in longitudinal epidemiological data (Hanson and Gluckman, 2008; Rutter, 2016; see also references in Table 1). This “archeology” (Hertzman, 2012, p. 17163) digs into different biological layers to ultimately identify differences at the level of DNA methylation or protein activity and gene expression. Although environmental approaches to embodiment assume that the incorporation or embedding of experience potentially takes place at multiple time-points, they often register only single events (preferably in early childhood) and their impact on a single biological level (e.g., the genetic and epigenetic level: Godfrey et al., 2007; Caspi et al., 2010). Only rarely, dynamic cross-level transformations of incorporated experiences are studied. This is mainly due to method- and data-related constraints. It implies, however, that we register only main tendencies and might miss most of the environmental impact and biologically embedding of dynamic experiences over the lifespan. In most cases, the “digging process” is limited to the final time-point and one biological condensate of interest.

In this way, environmental approaches often imply a direct causal link between an environmental event and a behavioral pattern, differences in psychological functions, or a mental health outcome. However, there is limited evidence for cases in which an input early in life directly, exclusively, and irreversibly affects the long-term outcome. More often we must assume that the biological foundation undergoes several dynamic developmental processes throughout an individual's life. Also, the mechanisms underlying acute activation of embodied experiences might differ from those grounding the long-term developmental pathways which channel the activation outcome. An experience may be embodied in a way that shapes a developmental pathway, which is then channeling but not determining the acute activity patterns, such as determining hormonal setpoints in the stress response system. This is critical, as any identified mediators and intermediate developmental stages of embodiment processes open up targets for interventions.

Table 2 summarizes developmental theories and their critical propositions for developmental embodiment research.

**Table 2:** Lifespan developmental theories and their critical propositions and implications for developmental embodiment research.

	**Critical propositions**	**Implications**	**References**
**Developmental systems theory**			Oyama, 2000; Bjorklund, 2003; Lickliter and Honeycutt, 2003; Overton and Lerner, 2012; Griffiths and Tabery, 2013
	*Developmental cascades:* • Capturing cumulative effects within a developmental pathway brought by the multiple interactions occurring in developing systems after an environmental input or another developmental event • Spreading across levels, among domains at the same level, or even across developing systems and generations	• Identify developmental cascades with cross-levels effects underlying embodiment phenomena	Masten and Cicchetti, 2010
	*Procedures:* • Referring to biological forms, structures, and patterns, but also chemical gradients etc. channeling the developmental process	• Embodiment phenomena result from an inherent parallelism of developmental changes and stability	Overton, 1991
**Gene-environment interaction models**			Hunter, 2005; Caspi et al., 2010; Esposito et al., 2018
	*Differential susceptibility hypothesis:* • Genomic information might not always directly affect the final phenotype but mediate the way, environmental influences and conditions get integrated during the course of development	• Genotype functions as embodied resource potentially shaping the degree to which environmental influences get incorporated during the life course	Belsky and Pluess, 2009; Pluess and Belsky, 2010
**Lifespan developmental psychology**			Baltes, 1987; Baltes et al., 2006; Li, 2006
	*Co-occurrence of gain and loss:* • Functional domains show different developmental trajectories as well as different ranges of changeability or plasticity	• Identify the mechanisms underlying processes of gain and loss at different biological level	Brandtstädter and Greve, 1994; Baltes et al., 1999; Staudinger and Baltes, 2001
	*Sociocultural-historical context and timeframe:* • Ontogenetic development as lifelong process of dynamic and selective adaptation based on the interaction of biological, cultural, and context factors	• Identify socio-cultural variation in developmental pathways with potential impact on incorporation and expression of embodied experiences	Baltes, 1987
**Lifespan perspective on motor development**			Thelen et al., 1987; Kamm et al., 1990; Schmuckler, 1993; von Hofsten, 2004; Haywood and Getchell, 2020
	*Rate limiters:* • In the interaction of individual, environmental, and task constraints during motor development, individual constraints at each system level can either support or hinder the development of new or the maintenance of existing motor skill	• Cross-level effects in the process of evolution and involution of motor capabilities underlying embodiment phenomena	Newell, 1986
	*Interrelationship of developmental timescales:* • Embeddedness of motor learning within the process of motor development and mutual responsiveness of both	• Consider different developmental timescales of processes underlying embodiment phenomena	Adolph, 2019

*A variety of developmental theories, models, and frameworks, targeting different system levels, provide conceptual and methodological foundations for an interdisciplinary framework, which integrates agency and environmental approaches to embodiment. The table provides an overview of these developmental theories, with their critical propositions and related implications for developmental embodiment research*.

## Developmental Embodiment Research: Cross-Disciplinary Examples

Practical examples of interdisciplinary embodiment research are still rare. Among these examples, only a few explicitly address embodiment processes from a developmental perspective. Here, we present a small selection of them, which shows the range of disciplines and research fields for which a developmental perspective of embodiment provides a useful platform. We selected these examples to illustrate the productivity but also some of the challenges of our proposed framework. Because of the many-faceted conceptualization of embodiment in the life sciences, the following examples can only be spotlights, highlighting different levels and areas of embodiment research, where, e.g., a developmental perspective is already employed, but needs refinement and more systematic standards across timepoints and experimental systems (see section Example 5: Epigenetic Mechanisms as Biomarkers for the Impact of Early Life Stress on Mental Health), or where such a perspective would help integrate data (see sections Example 1: Age-Related Cognitive Decline Impacts Motor Control and Example 4: The Developmental Impact of Limited Interoceptive Perception in Autism Spectrum Disorders) or provide additional criteria for competing theoretical explanations (see section Example 3: The Role of Sensorimotor Systems in Abstract Concept Representation and Example 7: Modeling Motor and Cognitive Development With Robots). Also, the examples described in the following demonstrate that the integration of timescales and levels needs to be case specific for each embodiment phenomenon, since, depending on the methods and data available, studying cross-level effects and indicators of long-term changes are quite different across disciplines and research topics.

### Example 1: Age-Related Cognitive Decline Impacts Motor Control

One first example, for how current interdisciplinary embodiment research profits from an explicit developmental perspective, is motor decision making. Decision making research has a long tradition in psychology. However, its relevance for understanding changes in movement coordination of everyday activities such as reaching and grasping has only recently been acknowledged: Cisek (2007), Cisek and Kalaska (2010), and Cisek and Pastor-Bernier (2014) highlighted the embodied nature of motor decision making, and its temporal dynamics during movement planning and control in a series of theoretical papers. Following this approach, Gallivan and Chapman (2014), Gallivan et al. (2018), Krüger and Hermsdörfer (2019), and Salzer and Friedman (2019) provided empirical evidence for these assumptions by showing changes in the execution of reaching movements under different conditions for motor decision making. It has been suggested that the perceived or expected biomechanical costs of a movement can reverse decisions to reach to particular targets (Burk et al., 2014) and can bias perceptual decision making when coupled to motor responses (Hagura et al., 2017). Thus, embodiment research, spanning the levels of neural, sensory, and motor activity, as well as complex psychological function and behavior, has advanced the cross-disciplinary understanding of decision making processes. Still, what is largely missing at present is the integration of empirical evidence on lifespan developmental changes of cognitive, perceptual and motor processes, stemming from the different research disciplines, into (motor) decision making theories: i.e., how age-related changes in cognitive and perceptual decision making, due to age-related changes in cognitive functioning and underlying neural networks (Mata et al., 2007; Eppinger et al., 2011; Kurnianingsih et al., 2015), relate to age-related changes in movement coordination and motor function (Verrel et al., 2012; Krüger et al., 2013), and vice versa. A developmental perspective on embodiment would allow for this integration by highlighting the dynamic and mutual interrelationship between motor and cognitive functioning across the lifespan, potentially also providing hints for the origins of the increasing inter-individual variability in cognitive and motor functioning with increasing age. In addition, it opens new avenues for research in the context of neurorehabilitation, since it underlines the need for multi-professional interventions to alleviate motor and cognitive impairments after e.g., stroke.

### Example 2: Motor Expertise Changes Perception and Cognition

In a different context, the interaction between motor skills, perception and cognition, and their neural basis is already studied from a developmental perspective, but on a much shorter timescale than lifespan development: In research on motor expertise, a developmental perspective has been adopted to measure how the adult brain changes during motor skill learning and physical training (Wenger et al., 2017). This approach of observing the dynamics and patterns of neuroplasticity during motor learning might contribute to explaining embodiment phenomena found in this context. Several studies convincingly showed that motor expertise changes perception and cognition. Movements which had been extensively trained were more readily recognized in subsequent visual discrimination tasks (Casile and Giese, 2006; Aglioti et al., 2008). Importantly, these perceptual improvements cannot be explained by visual experience alone, but suggest motor learning-induced plasticity in the sensorimotor system to affect perception and cognition as well. This is in line with several theoretical assumptions. One of them is the common coding account which considers overlapping representations of action and perception (Prinz, 1997). It also fits with the notion that neural networks for motor control have evolved to contribute to both motor actions and cognition (Ptak et al., 2017), which, again, is in line with a dynamic systems approach to cognitive development (Thelen, 2000) and with the predictive coding framework (Kilner et al., 2007). From this perspective, it is a pressing research question whether and how neuroplasticity in the sensorimotor system accounts for the development of special cognitive and perceptual skills in movement experts.

### Example 3: The Role of Sensorimotor Systems in Abstract Concept Representation

A developmental approach could also provide valuable new insights into the mechanisms underlying the representation of abstract concepts. In particular, by studying children's acquisition of abstract vocabulary we can test claims about the role of sensorimotor systems in knowledge representation (for a review see Pexman, 2019). Currently, at one end of the spectrum, amodal theories posit that knowledge is represented symbolically, which means that concepts are distinct from the ways we experience them (e.g., Quillian, 1969; Pylyshyn, 1985). At the other end of the spectrum, strongly embodied theories posit that knowledge is grounded in sensory, motor, and emotion systems (e.g., Glenberg and Gallese, 2012; Glenberg, 2015). Between these poles lie multimodal or hybrid theories, which posit that knowledge is represented in many ways (e.g., language, emotion, introspective, and sensorimotor) and that different kinds of information are important for different types of concepts (e.g., Barsalou et al., 2008; Borghi et al., 2019). These theories have been tested extensively in the context of research on adult concepts and language processing, with much recent support for multimodal theories (for a review see Zwaan, 2014). The underlying predictions about the acquisition of word meanings during language development in children have only begun to be tested (Wellsby and Pexman, 2014), with a handful of recent studies testing the validity and area of application of two competing theoretical proposals, the emotion bootstrapping proposal (Ponari et al., 2017, 2020; Lund et al., 2019) and the language competence proposal, suggesting that future studies should more carefully consider children's acquisition of different types of abstract words (Lund et al., 2019). These recent studies provided some initial insights, but their cross-sectional designs and methods offer limited inferences about children's representations of abstract concepts. Studies have not yet systematically explored the predictors and outcomes of abstract vocabulary acquisition.

### Example 4: The Developmental Impact of Limited Interoceptive Perception in Autism Spectrum Disorders

Selfhood and emotions have long been understood to be grounded in representations of the physiological state of the body (James, 1994; Damasio, 1999; Critchley et al., 2004; Craig, 2009b; Seth, 2013). More recently, research into interoception has demonstrated the extensive significance of our inner bodily signaling systems for decision making, time perception, emotion processing, and behavior in general (for review, see, e.g., Herbert and Pollatos, 2012; Seth, 2013). Consequently, for disorders that involve disturbances of self-representations (e.g., psychosis), emotional processing (e.g., alexithymia, anxiety and mood disorders), or with strong somatic components (e.g., depression and eating disorders), a primary dysfunction in the perception and regulation of body states has been considered (Paulus and Stein, 2010; Herbert and Pollatos, 2012; Stephan et al., 2016; Petzschner et al., 2017; Khalsa et al., 2018). An example are autism spectrum disorders (ASD), a spectrum of neurodevelopmental conditions characterized by lifelong difficulties in social and emotional functioning (among other impairments, Frith, 2014). ASD have been hypothesized to be related to interoceptive failure (Quattrocki and Friston, 2014). However, studies investigating interoceptive abilities in adults with ASD have yielded mixed results (Garfinkel et al., 2016; Shah et al., 2016; Gaigg et al., 2018; Palser et al., 2018). Critically, these discrepancies can potentially be resolved by adopting a developmental perspective: Quattrocki and Friston (2014) suggest that during a critical period of early childhood development, interoceptive signals need to be contextualized to support a typical development of emotional awareness and social attention. In particular, the association of interoceptive signals of warmth and satiety with an infant's caregiver serves as the basis for attachment behavior and endogenous social attention. The authors theorize that a difficulty in interpreting one's own bodily signals early on, potentially caused by abnormal regulation of the oxytocin system in ASD, prevents such associative learning between interoceptive and exteroceptive (social) cues, and leads to impairments of emotional awareness and social interaction later in life. Consistent with these ideas, recent studies support a diminished interoceptive accuracy in children with ASD (Nicholson et al., 2019), and specific impairments in the integration of interoceptive and exteroceptive information (Noel et al., 2018). However, data on interoceptive abilities in infants are scarce (although suitable experiments have been proposed) and very little is known about how these abilities develop across the lifespan (Murphy et al., 2017).

### Example 5: Epigenetic Mechanisms as Biomarkers for the Impact of Early Life Stress on Mental Health

One example for the potential but also the difficulties to identify links between different levels of embodiment when following an environmental approach is research investigating epigenetic mechanisms underlying long-term mental health effects of early life stress. Data from several longitudinal studies, clinical samples, as well as animal research support the link between early life stress and mental health (see the review by Provençal and Binder, 2014). Although some assume that the effects of early life stress only impact the individual after multiple stressful experiences (Binder et al., 2008; Danese et al., 2011; Zannas et al., 2015), there is consensus among researchers that early life stress gets somehow biologically embedded or embodied during critical periods in a way that mediates later effects. A growing body of research has identified stressor specific effects and effects of different stressor intensities (Bock et al., 2015; Lux, 2018; Aristizabal et al., 2019), as well as several factors of resilience or reversibility (Harris et al., 2016; Serpeloni et al., 2019; Hartmann and Schmidt, 2020, Francis et al., 2002). The main challenge for the research field is to determine the causal pathway that integrates effects of different embodiment levels (from molecular to behavioral and cognitive) at several critical time points across the lifespan, and especially in early life periods.

Here, one line of research focuses on epigenetic modifications as relatively stable intermediate level, coordinating genetic constitution, and environmental signals, as indicated by animal studies (Weaver et al., 2004; Murgatroyd et al., 2009; Franklin et al., 2010). Although epigenetic modifications following early life stress are reported repeatedly (for a review see Aristizabal et al., 2019; with focus on human studies Vaiserman, 2015), mechanistic links between these and other levels of embodiment including the stress hormone system and stress and emotion regulating neural networks are still not identified (Aristizabal et al., 2019). Instead, contradictory findings complicate the picture. To make sense of the current findings, the field works at developing integrative models spanning different time-points of embodiment and highlighting critical periods, during which exposure to adverse environments and stress impacts developmental pathways much stronger than during other periods over the lifespan (Lupien et al., 2009; Bock et al., 2015; Non et al., 2016; Non, 2021). In addition, more research is needed to distinguish between long-term epigenetic modifications observed following early life stress and those related to acute stress exposure later in life, to determine the importance of developmental timing and cumulative effects. Trans- and inter-generational effects in mammals and humans are implicated but very challenging methodologically to determine, and thus still under controversial debate (Horsthemke, 2018; Lacal and Ventura, 2018; Perez and Lehner, 2019).

### Example 6: Identifying Sensitive Periods for the Incorporation of Embodied Experiences

An example of research that could address the question of critical periods using a developmental framework are experiments that can establish causation and go beyond traditional observational studies. For example, Provençal et al. (2019) tested epigenomic effects of exposing fetal-derived neurons *in vitro* to stress hormones (glucocorticoids) across different time periods of exposure, and even how they prime future gene expression responses to stress. While experiments like these are divorced from interactive effects in the body, they can be a first step toward establishing if and when critical periods for stress exposure may alter the epigenome in the relevant tissue of interest. These experiments, of course, test only short-term early life embodiment and would benefit further by integrating findings with longitudinal human studies to see if the same epigenetic effects last throughout the life-course across accessible tissues and contribute to long-term mental health effects. For this, the research field would clearly profit from knowledge about the developmental dynamics of embodiment processes. Although epigenetic mechanisms are still the primary focus, more complex cross-level effects of stabilization and mediation, especially between epigenetic modifications, the stress hormone system, and the formation of neural networks have been identified as promising targets for this line of research (Lux, 2018; Aristizabal et al., 2019; Fogelman and Canli, 2019, Hartmann and Schmidt, 2020).

### Example 7: Modeling Motor and Cognitive Development With Robots

In the field of human robotics, the turn to embodied cognition theories initially went against the information processing paradigm of artificial intelligence (AI) (see Hoffmann and Pfeifer, 2018). Until then, AI units constructed according to the information processing paradigm showed tremendous success in pattern recognition and human-like learning behavior, but they were strongly limited by the available processing capacity. The turn to embodied cognition theories within robotics was supported by the production of simple robots imitating the anatomy of living organisms and showing stable and sophisticated motor and sensory behavior without the need of complex information processing systems. One of the most striking examples is the passive dynamic walker, inspired by the anatomy of human legs, which is able to walk stably and smoothly on a plain surface due to its mechanical properties and without complex processing of movement control (McGeer, 1990; Collins et al., 2005). In a similar way, Brooks developed robots with simple parallel sensory processing units, partially hierarchically clustered, which navigate successfully within their environment without the need of complex representation (Brooks, 1991). However, these example robots, as impressive as they are, are restricted to on-time processing of the ‘here and now' and not able to learn from previous experiences. A developmental perspective on embodiment, combining the embodied cognition approach with machine learning and similar algorithm-based self-controlled processing shows promising potential for overcoming some of the boundaries inherent to each approach (Bongard et al., 2006; Sloman, 2009; Hoffman, 2012; Hoffmann and Pfeifer, 2018), with first computer models (Hoffman, 2012) and prototypes being developed. These robots use mechanically inbuilt embodied information to guide motor control and sensory input to reduce processing capacities necessary for interactions with the environment, which are then free for complex, i.e., capacity demanding cognitive processes, e.g., learning.

In addition, human robotics research based on a developmental embodiment perspective also provides a platform and model for the study of human cognition and motor development by allowing to control and observe the functional elements as well as the developmental processes to a degree which is not possible in living organisms (Pfeifer and Bongard, 2006; Hoffmann and Pfeifer, 2018). For example, based on a computational approach to developmental systems neuroscience, Schöner et al. (2018) simulated a simple neural dynamic model of movement generation, serving as platform for discussing infants' developmental challenges as they learn to reach for objects. Also, based on this model, they were able to construct a neural inspired robot imitating the neural processes underlying the reaching behavior (Tekülve et al., 2019). Although such modeling approaches are limited to engineering and computing capacities, they provide a potent tool to test hypotheses between different levels of embodiment, especially between the neural network level and the level of sensorimotor processing and motor actions.

## Toward an Integrative Framework for Developmental Embodiment Research

As the previous examples show, developmental embodiment research focuses on cross-level effects underlying developmental processes, and a lifespan perspective to overcome restrictions of previously used approaches within their disciplines. An interdisciplinary framework acknowledging this research approach has the power to provide a matrix and platform for specific empirical studies or experiments on embodiment processes and phenomena, executed based on discipline specific standards and methods. Such a framework, as we propose in the following, maps out connections between levels of embodiment, with the goal to identify and address white spots across the map which, when filled, further complete the picture of a specific embodiment phenomenon. In this section, we will first introduce the main pillars of our framework and then outline important steps to bridge different timescales and levels of embodiment in cross-disciplinary embodiment research.

**Figure 1:**
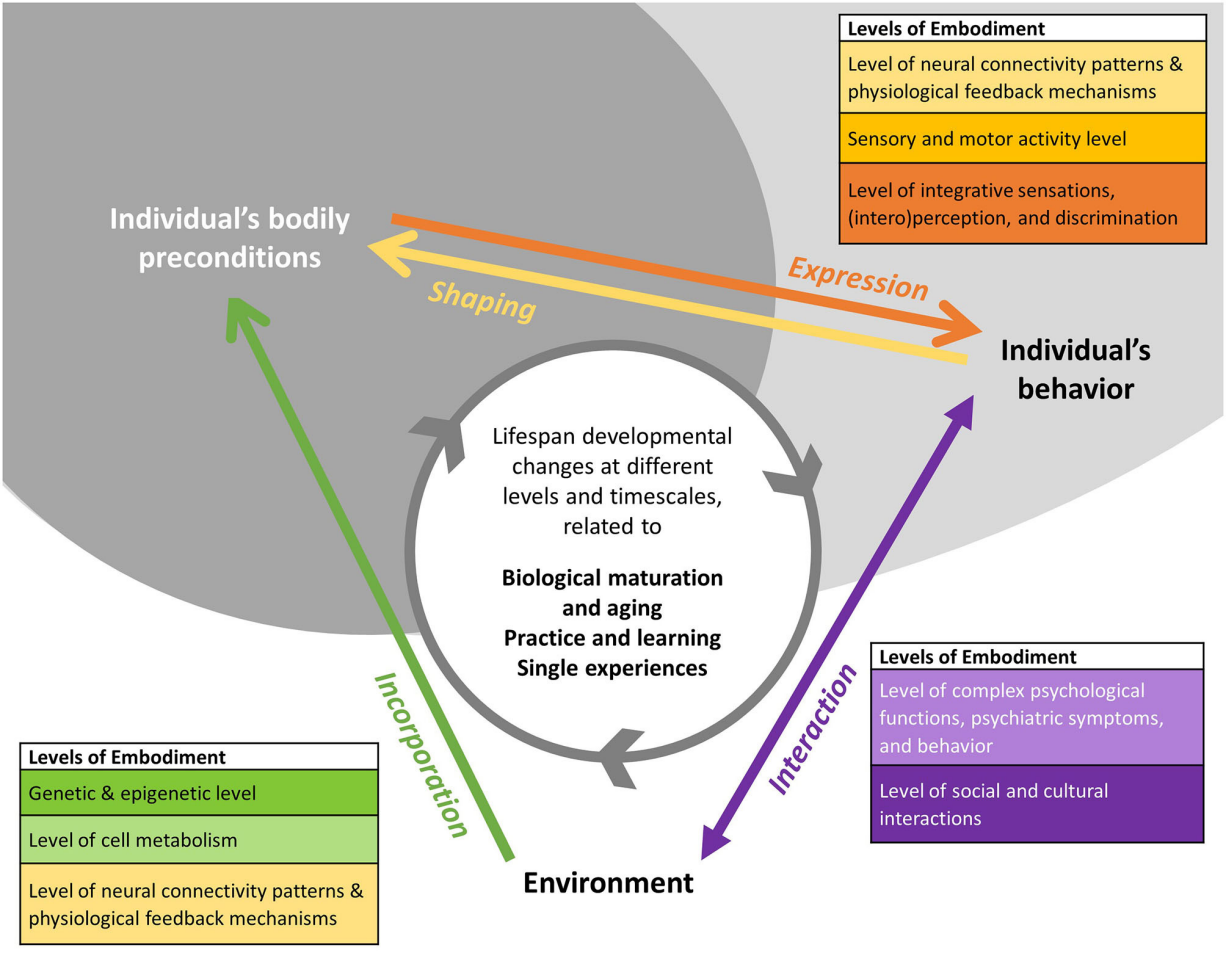
Developmental perspective on embodiment. The figure illustrates how different conceptualizations of embodiment relate to each other from a developmental perspective. Arrows represent the different embodiment processes with the colors indicating the physiological and functional levels at which they are analyzed. The direction of the arrows indicates the perspective on the person-environment relationship. While *environmental approaches* focus on the process of incorporation of experiences over the lifespan (green arrow), *agency approaches* focus on the process of mobilization of embodied experiences and knowledge (purple arrow) within a specific behavior, emotional state or functional ability, simultaneously expressing and, through the action etc., shaping bodily preconditions (orange and yellow arrow). The developmental perspective emphasizes that both types of processes change the person-environment relationship. Lifespan changes of bodily preconditions, occurring at different timescales, result in lifespan developmental changes of embodiment phenomena and processes (gray colored circular arrow).

*Environmental and agency approaches represent complementary perspectives*. As Figure 1 illustrates, environmental and agency approaches to embodiment represent complementary perspectives in this endeavor: While environmental approaches focus on the process of *incorporation* of experiences over the lifespan (green arrow), agency approaches focus on the process of *expression* of embodied capacity within a specific behavior, emotional state or functional ability, simultaneously expressing and, through the action etc., *shaping* bodily preconditions (orange and yellow arrow). Both complementary, but analytically distinct approaches are interconnected by developmental processes along the individual's developmental timeline (gray-colored circular arrow).

*Multi-level approach*. In addition, Figure 1 illustrates the different physiological and functional levels potentially involved in the processes of incorporation, shaping and expression of embodied experiences. At the moment, these levels are often studied separately in different disciplines and by different theoretical schools, depending on their respective concept of embodiment. An explicit developmental perspective, relying on critical propositions of developmental theories, shifts the focus on the developmental processes interconnecting the mechanisms underlying a specific embodiment phenomenon at the different levels.

For example, from a developmental embodiment perspective, epigenetic modifications as well as the underlying genotype could be understood as developmental resources. These resources shape the way and degree to which environmental influences are incorporated during the life course and impact developmental outcomes, cumulating, for example, in disease vulnerability or resilience. These then represent some of the underlying bodily preconditions, for example, in a situation of acute mental distress, potentially shaping the symptomatic re-/action to a stressful situation. Pluess and Belsky (2010) discussed these observations as *differential susceptibility* to environmental signals (see Table 2) and *environmental sensitivity* (Pluess, 2015), Weaver et al. (2004) framed it as *epigenetic programming*. The impact of molecular changes at the epigenetic level on the adult phenotype, including disease symptoms and responsiveness to therapy, is a pressing research question. Specifically, at the genetic and epigenetic level (greenish level in Figure 1), environmental epigenetics (Weaver et al., 2004; Zhang and Meaney, 2009; Bollati and Baccarelli, 2010) and studies of gene-environment interactions (Caspi et al., 2003, 2010; Risch et al., 2009; Culverhouse et al., 2018) analyze molecular long-term (ontogenetic) effects of embodied experiences. In addition to one-time severe impacts, such as traumatic events or toxin exposure, also multidimensional and enduring environmental signals are studied, such as enriched environments (Zhang et al., 2018), bullying (Mulder et al., 2020), racial discrimination (Brody et al., 2016), socio-economic status (Needham et al., 2015; McDade et al., 2019), social deprivation (Non et al., 2016), and gender experiences (Cortes et al., 2019). For these fields, our framework would provide the currently missing cross-disciplinary matrix to integrate the different time-points and levels under study.

*Accounting for change and stability*. Further, from a developmental embodiment perspective, we conceptualize embodiment phenomena as resulting from a balance of developmental change and stability, which depend on the presence, strength, and timing of the environmental signal. Coming from Developmental Systems Theory, which emphasizes that the developmental system consists of dynamic feedback loops, Overton (1991) introduced the term *procedures* to characterize temporarily stabilized parts of the developmental systems present in embodiment phenomena (see also Table 2). Bones, grown structures and patterns of tissue, muscle constitutions and setpoints within metabolic or hormonal feedback loops, neural pathways and networks, epigenetic modifications but also automized behavioral patterns potentially function as established and stabilized procedures sculpting developmental pathways. Although procedures continuously need to be maintained within the developing system, we can classify them as embodied when the energetic costs to transform a procedure outweigh those to stabilize it, keeping in mind that the balance between energetic costs for stabilizing a procedure and transforming it might change during the course of the lifespan.

For example, the hormonal feedback loops underlying the stress response are established early in development. During this critical developmental period, set points of up- and downregulation of the stress response in presence of an acute stressor are established depending on the individual metabolic conditions and stress experiences during this period. These are then stabilized, however, the stabilization might be challenged by following life experiences, such as traumatic events, hormonal imbalances during adolescence, pregnancy, chronic stress, or aging, which all might lead to changes of the original set-point. The exact mechanisms of stabilization and change of these set-points are still only partially known, yet, it is assumed that they contribute to stress-related mental health impacts.

Another example would be establishment of neural pathways underlying automatized word recognition in reading. The ability to learn how to read depends on age-dependent neural and cognitive development with only some degree of inter-individual variability indicating dependence on other bodily embedded developmental processes. Once the basic ability to learn how to read is developed, instructions and practice are needed until the stage of automized word recognition is reached. The neural mechanisms, such as synaptic sensitization and neural pathway stabilization, underlying this automatization process are likely similar to (and may even rely on) processes of automatization of other motor and sensory activities, yet, again, they are still partially known. Their further elucidation would contribute to our knowledge on the mechanisms underlying various variants of dyslexia as well as possible impacts of strokes and subsequent rehabilitation therapy.

Developmental embodiment research would address these mechanisms with a multi-level cross-disciplinary approach and lifespan perspective. It would also address that the pathways of embodiment are then potentially two-fold, as can be traced in Figure 1, via a more passive incorporation (green arrow) or via the expression-shaping cycle (yellow and orange arrows) mediated by the individual's interaction with the environment (purple arrow). As both are depending on each other, with acute activation necessary for change and maintenance on the one side and incorporation setting some of the preconditions for the activation process on the other side, overcoming the current practical divide in embodiment research between environmental approaches and agency approaches is crucial to further advance our understanding of these processes. Interconnecting them via a developmental perspective would allow to assess criteria and thresholds for change and stability at a specific level (of, for example, genetic and epigenetic activity, cell metabolism incl. hormonal activity, neural network activity, sensory, and motor activity) within different developmental periods across the lifespan.

*Cross-level effects cumulating in developmental cascades*. An explicit developmental perspective on embodiment, building upon critical propositions of developmental theories, also highlights the cross-level dependencies and effects of embodiment processes and phenomena. According to the life span perspective on motor development (see, for example, Haywood and Getchell, 2020) skilled motor behavior follows from a self-organization process of multiple system levels within an individuum, e.g., muscular, skeletal, neural, cognitive, etc., referred to as “individual constraints” (Newell, 1986). Based on this assumption, it follows that, for developmental motor behavioral changes to occur, all individual constraints have to be developed to a required level. Here, the proposition that individual constraints can act as *rate limiters*, i.e., either restricting or facilitating the development or decline of motor skills, implies cross-level dependencies of development (see also Table 2). Masten and Cicchetti (2010) discuss such cross-level effects under the term *developmental cascades* (see Table 2). Developmental cascades capture cumulative effects within a developmental pathway brought by the multiple interactions occurring in developing systems after an environmental input or another developmental event. The key characteristic of such a cascade is that the effect spreads across levels, among domains at the same level, or even across developing systems and generations (Masten and Cicchetti, 2010, p. 492). From the perspective of developmental embodiment research, it would then be the goal to identify developmental cascades with cross-levels effects underlying embodiment phenomena. This also stipulates identification of levels involved and affected within a specific embodiment phenomenon, identification of transmission hubs between these levels as well as identification of time-points of transmission. These differ for the particular phenomenon under study, e.g., for the biological embedding of early adversity and its long-term impact on mental health, the way motor experiences ground the development of cognition and emotion processing, and how imagination techniques improve motor rehabilitation outcomes after a stroke.

*Identification of transmission hubs*. Trying to identify developmental cascades when taking the systemic and dynamic character of biological processes seriously, as from a developmental systems perspective, would require to account for all levels of the system at every time-point during development. However, such a comprehensive approach is not feasible in concrete empirical studies, even with cross-disciplinary collaborations addressing different levels, timescales, and data modalities. Therefore, we propose to focus on the identification of transmission hubs between levels of embodiment. Transmission hubs constitute the molecular, physiological, cognitive, etc. structures which participate in the transmission of signals related to a particular embodied experience across levels, such as, e.g., neural networks underlying motor execution that are also involved in imagination and cognitive processing, neural and hormonal correlates of interoception in emotion processing and related disorders (see section Developmental Embodiment Research: Cross-Disciplinary Examples, Example 4), molecular feedback loops underlying the regulation of synaptic plasticity, neurotransmitter activity, and the stress response, as well as processing of biomedical knowledge influencing self-perception, symptomatic experiences, and mental health. These transmission hubs are characterized by their critical role to enable or block transmission of developmental changes between levels, thereby stipulating and canalizing embodiment processes. Importantly, with respect to the developmental perspective, transmission hubs are not fixed across the lifespan, but change due to developmental processes, e.g., in the degree of plasticity, the involved biological, and functional levels of transmission, the signal intensity needed to induce transmission. They are also likely involved in the constitution of critical and sensitive periods but not limited to them.

*Accounting for gain and loss*. From research in the perspective of lifespan developmental psychology, we draw on the notion that gain and loss always occur together in ontogenetic development (Baltes, 1987) for our integrative framework for developmental embodiment research (see also Table 2). Thus, in addition to analyzing growth and maintenance as well as recovery and resilience, studying the regulation of loss is equally important. While lifespan developmental psychology focuses on the developmental consequences of this selective channeling process, developmental embodiment research additionally aims at identifying the mechanisms underlying these processes at different biological levels.

*Functional domains differ in their developmental trajectories*. Also in reference to research in the perspective of lifespan developmental psychology, we account for the notion that different functional domains have different developmental trajectories across the lifespan, which also differ in their range of changeability or plasticity and even between individuals with comparable functional outcome (Brandtstädter and Greve, 1994; Baltes et al., 1999; Staudinger and Baltes, 2001). As a methodological consequence, person-centered (holistic) and function-centered approaches need to be combined to study these developmental trajectories (Baltes et al., 1977; Baltes, 1987). Although a function-centered approach will probably be used most in developmental embodiment research, a person-centered approach might become more relevant when the field expands into translational research and when focusing on inter-individual differences in the expression of embodiment phenomena.

*Accounting for sociocultural-historical changes*. Finally, a developmental embodiment perspective reinforces the necessity to account for sociocultural-historical changes and their impact on the bodily preconditions of psychological functions. This includes the effects of socio-cultural contexts (family, school, work, etc.), historical changes of educational and professional systems, cultural norms, (religious and other) traditions, as well as historic events, such as war periods and collective traumatic events. Some examples following an environmental approach to embodiment are efforts to identify epigenetic mechanisms underlying the long-term impact of traumatic events such as the attacks of 9/11 (Kuan et al., 2017) or war crimes (Ramo-Fernández et al., 2015). Another example in this perspective are studies on changing neural networks and cognitive performance in children after entering school (Brod et al., 2017). Examples following an agency approach to embodiment are analyses of bio-looping effects of cultural knowledge and resources including scientific concepts and how they influence the active construction of self-perception patterns and identity, emotion regulation, and health and body related behavior (Seligman et al., 2015; Kirmayer and Gómez-Carrillo, 2019).

Thus, for developmental embodiment research, considering lifespan dynamics of embodiment does not only entail the consideration of different timescales of development, but also the consideration of different processes, which contribute to these developmental changes and the different timescales at which these processes act. Reconstructing the developmental cascade therefore requires integrating data generated with different experimental settings and study designs, at different time-points, at different levels, and across the involved disciplines.

### Bridging Timescales of Embodiment

With our framework for developmental embodiment research, we explicitly aim at bridging the different timescales of embodiment focused on either by agency or environmental approaches (see Figure 1).

*First*, we emphasize studying developmental processes related to embodiment phenomena along different timescales, foremost across the whole lifespan. Due to the underexplored lifespan perspective, buffering effects and functional changes during development are often underestimated, as shown, for example in research on the biological correlates of resilience (Feder et al., 2009) and longitudinal studies of stress buffering effects of social support and mental function in old age (Toyama and Fuller, 2020). To identify these, the study of functional gains should be complemented with the study of mechanisms of maintenance and of the regulation of loss, as well as with the search for alternative developmental pathways in studies using inter-individual comparisons. However, shorter timeframes of developmental dynamics also have to be considered as periods in which such alternative developmental pathways are initiated. When considering development as a result of interacting constraints at different system levels within the individual, and between the individual and the environment, single events, as, for example, traumatic experiences or learning processes may lead to immediate changes of developmental pathways, with lifelong consequences for related embodiment phenomena. Thus, depending on the phenomena under study, also shorter timeframes of analysis, e.g., for specific learning processes, or a repeated analysis of shorter timeframes spread out across the whole lifespan, as often used in longitudinal studies, should be considered for being able to identify buffering effects und functional changes across the lifespan.

*Second*, despite a focus on the whole lifespan, we also need to continue identifying sensitive periods during which experiences are more likely to be embedded. For this, we need to consider that sensitive periods differ across different neural and other physiological systems contributing to motor, cognitive, and brain development. Moreover, the study of sensitive periods, such as the perinatal phase, early childhood, and adolescence, needs to be complemented by the study of subsequent stabilization periods, and phases of recession and degeneration in later life. Only when the embodied experience, as acquired through interaction with the environment, is preserved, can it be relied on at a later time-point and can thus have a developmental impact. These stabilization processes might provide promising targets for intervention, especially with regard to later phases of accelerated decline in functioning at multiple behavioral and physiological levels.

*Third*, for every behavioral outcome, symptom, or function studied in terms of embodiment, commonalities but also differences between the biological processes contributing to the incorporation of experiences and those used for expressing the embodied experiences during action need to be taken into account. This is of particular practical relevance for interventions, which may differ significantly in their structure and outcome depending on whether they target the first or the latter. For example, in the context of mental health, an intervention might aim at increasing resilience (to prevent incorporation of negative experiences), or it might aim at increasing behavioral flexibility and at re-learning after a negative environmental impact (such as cognitive-behavioral interventions), or at interfering neurochemically with the circuit that implements the embodied processes (as pharmacological interventions do).

*Fourth*, depending on the system level, an evolutionary timeframe needs to be considered. Identification of phylogenetic evolved plasticity and environmental sensitivity parameters for specific target systems and tissue will inform clinical and intervention studies. However, it is important to not confuse the phylogenetic timeframe, which addresses species development at the population level in a co-developing organism-environment context, and the ontogenetic timeframe within the lifespan of an organism. Developmental dynamics of different physiological systems or neural networks will vary between individuals. Identification and description of species-specific developmental periods needs to account for this variability.

*Fifth*, across these different timescales of phylogenetic developed sensitive periods, their socially and culturally shaped realization during ontogenetic development, and such single short-term experiences with long-lasting effects, we suggest focusing on the transmission hubs underlying developmental processes, which connect processes and functions at different levels with each other and provide transition points within the developmental pathways. One example would be the study of traumatic stress during sensitive periods of brain development contrasted with traumatic stress experienced before or after such a sensitive period. The transmission hubs of interest here would be those hormonal, neural, and molecular feedback loops whose interaction constitute sensitive periods thereby opening up the involved systems for the embodiment of the environmental signal.

### Bridging Levels of Embodiment

In addition to accounting for different timescales that underlie the processes of incorporating and expressing embodied experiences, we propose that a developmental perspective allows for connecting different biological and functional levels of embodiment. For that, the involved levels of embodiment have to be clearly differentiated to carve out conceptual and methodological gaps that need to be bridged when studying a specific embodiment phenomenon. Identification of levels is a precondition for cross-disciplinary data integration.

There are multiple ways to differentiate levels of embodiment. For the aim of our proposal—connecting different approaches of embodiment research from a developmental perspective—we differentiate between levels along the methods used to assess, observe, induce, and evaluate changes of embodiment. This is a first step in the process of integrating data from different research fields and approaches with each other, despite the conceptual variability in embodiment definitions.

We propose to differentiate between at least seven levels of embodiment (see Table 3): The genetic and epigenetic activity level (1), the level of cell metabolisms (including proteomics analyses) and single neuronal activity (2), the level of neural connectivity patterns and physiological feedback mechanisms (e.g., hormonal, metabolism related) (3), the sensory and motor activity level (4), the level of integrative sensations, (intero)perception, and discrimination (5), the level of complex psychological functions, psychiatric symptoms, and behavior (6), and the level of social and cultural interactions (7). The number of levels and their distinction are not exclusive. We explicitly encourage understanding them as to be open to adaptations depending on their worth for the respective empirical study and the development of new methods.

**Table 3:** Levels of embodiment, types of data, and biological materials or (bio-)social systems, matched with embodiment concepts by which they are addressed.

			**Embodiment concepts**								
			**Embodied cognition**	**Embodied simulation**	**Somatic marker hypothesis**	**Inference-control loop**	**Biological embedding of experiences**	**Environmental epigenetics**	**Developmental origins of health and diseases**	**Phenomenological approaches to embodiment**	**Bio-looping**
**Level of Embodiment**	**Type of data**	**Biological material/(bio-)social system**									
Genetic and epigenetic level	DNA sequence (genetic polymorphisms), RNA expression levels, gene × environment interactions, DNA methylation patterns, histone modifications, quantification of microRNA	DNA, mRNA, ncRNA (incl. different types of microRNA), DNA methylation, chromatin structure									
Level of cell metabolism	Protein level quantification, single unit-recordings, cell anatomy measures (size, form, type, count)	Cell specific proteome, synaptic sensitivity, firing rates, cell anatomy									
Level of neural connectivity patterns and physiological feedback mechanisms	EEG, fMRI, resting state MRI, hormone levels, diverse measurements of basic metabolic functions (e.g., heart rate, breathing, blood glucose levels)	Neural network activity, hormone levels, physiological feedback cycles									
Sensory and motor activity level	EEG, fMRI, behavioral observation of movement patterns, reaction time to sensory stimuli	Motor action, sensory function, neural activity in sensory and motor systems									
Level of integrative sensations, (intero)perception, and discrimination	EEG, fMRI, experimental tests of perception and basic cognitive functions (e.g., via reaction time, stimulus intensity, conflicting stimuli)	Sensory integration, basic cognitive functions, basic levels of self-awareness, pain perception									
Level of complex psychological functions, psychiatric symptoms, and behavior	Behavioral data (field observation, experimental induction), psychological and psychiatric diagnostics (test, interview), self-reports, introspection, intersubjective communication, health records	Psychosocial and physical health, first-person experiences, behavioral patterns (habits), complex cognitive functions									
Level of social and cultural interactions	Qualitative interview data, discourse analysis, behavioral data (field observation), socio-economic data, epidemiological data (e.g., prevalence rates, survival rates)	Socio-cultural interactions, complex behavior, intersubjective coordination and communication									

*The table provides an overview about the different levels of embodiment and by which embodiment concepts they are approached. Levels of embodiment are assigned to embodiment concepts according to the type of data and the biological material or (bio-)social system addressed by the embodiment concept. Blank boxes indicate conceptual and methodological gaps between concepts within a certain level (left to right) and between levels within a concept (top to down). The addressed levels of embodiment within an embodiment concept might change due to the development and use of new methods. The matrix allows one to spot potential cross-level collaborations across embodiment concepts, but also gaps indicating the need for further research to bridge levels of interest as well as large scale methodological constraints. Each color highlights a different level of embodiment (see also Figure 1)*.

We further propose to understand these levels as analytical tools rather than empirical entities, with the purpose to identify key processes, which coordinate and translate different biological mechanisms underlying embodiment as well as psycho-social and cultural interactions related to them. The method-based differentiation of these analytical levels also enables to visualize where data acquisition at different levels may be easily combined or exclude each other (for neuroscience/fMRI see Soares et al., 2016; for genomics/proteomics see Tyers and Mann, 2003; Manzoni et al., 2016; Vitrinel et al., 2019). Also, methods which describe embodiment and embodied experiences from different epistemological standpoints that do not map easily onto each other, such as cultural analysis, introspection, and the detection of neural activity or gene expression patterns, can be made explicit in this framework (for an example on embodied memory and social skills see Fuchs, 2016a). This supports the planning process of collaborative research projects spanning across levels.

The variety of methods used to differentiate between levels of embodiment may seem overwhelming to approach from the perspective of single research groups. Moreover, technical restrictions in data acquisition and epistemological gaps between data modalities (e.g., between subjective experience data and neurophysiological data) mean there will always remain blank spaces in the picture. However, we believe that the differentiation between levels provides a clear basis for trans-level collaboration between two or more research groups and even for larger research consortia. In any case, it helps to identify the blank spaces for specific cases of embodiment phenomena. Furthermore, it allows to spot potential transmission hubs between levels of investigation. These transmission hubs are important targets to understand the process of translating incorporation into expression of embodied experiences across the lifespan. Ultimately, they also constitute promising points of intervention.

Bridging different levels of explanations (and data modalities) is a challenge that is inherent to many fields in the life sciences. A popular tool for achieving such bridges is computational modeling. Formal models of processes at different levels and their interactions allow for an *in-silico* testing of how changes at one level translate into changes at another level. One recent example is the nascent field of computational psychiatry (Huys et al., 2016; Redish and Gordon, 2016), which acknowledges the multi-leveled nature of psychiatric diseases—ranging from the genetic and molecular level, neural circuits, cognition and behavior to the social and even cultural level—and attempts to bridge these levels of analysis using mathematical tools. The even more recent subfield of computational psychosomatics explicitly considers the role of body perception (interoception) and regulation for the understanding and treatment of mental diseases (Petzschner et al., 2017, 2021). Modeling homeostatic setpoints and their interactions with cognition provides a new framework for understanding the interconnectedness of bodily and mental well-being that becomes obvious in the symptom profiles of all psychiatric and psychosomatic diseases. More generally, these approaches hold great potential for studying embodiment phenomena across different levels.

### Clarifying Disciplinary Boundaries and Discipline Specific Criteria in Conceptualizing Embodiment and Embodied Experiences

One of the foremost practical challenges for integrative embodiment research, as suggested in the previous section, is that for each scientific discipline, there are different criteria for when an experience counts as embodied according to the different detection methods and data modalities. In addition, these criteria are sometimes ambivalent and need to be the subjects of further conceptual debates. Consequently, these discipline-specific criteria must be clarified for being able to relate different levels of embodiment and, consequently, embodiment approaches to each other.

At the genetic and epigenetic activity level, for example, we could ask whether detection of a functional relevant genetic mutation or its transcription in the target tissue, as indicated by gene expression analysis, represent the same or two different indicators of embodiment. When using epigenetic data, criteria are even more variable, including not only potential quantitative differences of functionally relevant modifications across tissue types but also different epigenetic mechanisms (DNA methylation, histone modifications, RNA interference etc.), which are functionally and hierarchically intertwined. In addition, epigenetic modifications are often evaluated as functionally relevant when they affect gene expression, although the relationship between gene expression patterns and, for example, DNA methylation is still not fully understood (Lea et al., 2018), and there may be a lag in timing before the functional effect is expressed. Thus, criteria for assessing embodiment can be too strict and not accounting for indirect as well as long-term effects of embodied experiences (Aristizabal et al., 2019), even within the research discipline.

For the level of neural activity, neural activation of motor areas either at a single neuron level or at the network level is often used as a criterion of embodiment, for example, in the detection of the fiercely debated “mirror neuron systems.” Here, the activation of motor neurons during the performance as well as the perception of a motor task is used as indicator of an embodied inner simulation or immediate representation of the task (Rizzolatti et al., 1996; Rizzolatti and Craighero, 2004). From a developmental neuroscience perspective, the developmental stage of a neural network, as indicated by age-specific connectivity patterns activated in a behavioral task, also functions as criterion for the type and degree of embodied experiences (Decety and Michalska, 2020). In contrast, for the level of emotion processing and interoception, Damasio et al. (2013), first referred to a single case lesion study only showing the anatomical absence of specialized neural networks as indicator that our emotion processing abilities are grounded in bodily processes. More recently, the interaction of interoceptive and emotional states as well as with perception and cognition is investigated more systematically by testing the impact of visceral signals and their neural processing on emotional, perceptual, and cognitive functions, and vice versa (for overviews, see Critchley and Garfinkel, 2018; Azzalini et al., 2019).

For the level of complex psychological functions, psychiatric symptoms, and behavior, Needham and Libertus (2011), discussing embodiment research in the field of cognitive development, refer to the experience of acting as a main criterion for embodied experiences. Based on studies conducted by Adolph (1997, 2000), which indicate that infants do not transfer their knowledge of surface characteristics, acquired through motor experience in one type of movement (e.g., sitting), to another type of movement (e.g., crawling), Needham and Libertus (2011) conclude that the information about surface characteristics is embodied via motor experiences and not via an abstract cognitive generalization of these characteristics. Furthermore, they illustrate the asynchrony of motor and cognitive development with different results of Piaget's famous cognitive development test of object permanence (the A-not-B error task) based on the behavioral data used to measure the response (gaze vs. pointing/grasping, in 3 to 4 vs. 8 to 10-month-old infants, respectively). Needham and Libertus (2011) argue that, at this early age, the motor experience of pointing and grasping is not yet connected to the visual detection of object permanence indicating different developmental pathways. Most importantly, the different criteria at the behavioral level for different age groups (pointing/grasping vs. gaze) reveal the importance and productivity of a developmental perspective for detecting the role of embodied experiences and the degree of embodiment underlying the cognitive functions under study.

Further, for higher psychological functions such as imagination, associative thinking, and language processing, Körner et al. (2015) introduce three different mechanisms underlying embodiment effects: *direct state induction*, indicating a direct impact on how humans feel or process information without interference of any other cognitive mechanism, *model priming* referring to changes in the accessibility of concepts associated with a bodily state, and *sensorimotor simulation* indicating mechanisms which affect the ease with which congruent relative to incongruent actions are performed. Furthermore, they outline a set of conditions to test which of these mechanisms are involved in an embodiment phenomenon under study (Körner et al., 2015). For example, when the fluency of two competing tasks, which use the same sensorimotor resources, is enhanced by untraining a more fluent action (as in a right- vs. left-handed task), the underlying embodiment mechanism is based on sensorimotor simulation and not modal priming. As elucidating as this is at the level of higher psychological functions, how can we identify tasks, which use the same sensorimotor processes, in the first place (beyond right- vs. left-handed tasks)? This knowledge necessarily precedes the psychological testing conditions. Furthermore, how do we differentiate between these mechanisms when they co-occur in the studied embodiment effect? From a developmental embodiment perspective, a first step could be to trace the occurrence of these different mechanisms during ontogeny as well as to identify the potentially varying underlying neural networks.

Last, at the level of psychiatric symptoms, embodied experiences of e.g., traumatic events, or of depressive or psychotic episodes are indicated by physical symptoms and symptoms of somatization, such as pain, phantom sensations, but also hormonal imbalances and increased inflammation markers (Schnurr et al., 1998; MacLachlan, 2004; Roh et al., 2007; Abbey et al., 2011; Goldsmith et al., 2012; Blumberg and Dooley, 2017; Yuan et al., 2019).

In sum, these examples for different criteria of embodiment at different levels of investigation echo the diversity and variability in concepts of embodiment and conceptualizations of embodied experiences across scientific disciplines. Thus, it is important to clarify the criteria of embodiment for each level and discipline when conducting embodiment research. The examples also show that embodiment is often detected by studying cross-level, and, by such, cross-disciplinary effects (e.g., between motor behavior and neural activity level). Moreover, they demonstrate that the actual task is to relate the effects at each level to each other. This is especially relevant for those innovative cross-disciplinary research areas that study the processes of incorporation and expression of biological embedded experiences across system levels, e.g., in the context of cognitive development, psycho-social well-being, language acquisition and processing, preservation and rehabilitation of cognitive and motor function, and many more.

## Conclusion

Embodiment research is at a turning point. The growing amount of data from various studies across a wide range of disciplines and theoretical schools, investigating embodiment phenomena and their role especially in mental processing and functions, highlights the need for an interdisciplinary framework of embodiment research. Innovative research areas such as movement psychology, social and developmental neuroscience, computational psychosomatics, social and behavioral epigenetics, human-centered robotics, and many more, that are facing issues of data integration across different levels of embodiment, would profit tremendously from such a framework. Especially the integration of behavioral data with data from different biological levels, each of which depend on their own developmental timescales and dynamics, is challenging for these fields. In addition, there is a growing need for a cross-disciplinary consensus on level-specific criteria of embodiment. We propose that a developmental perspective on embodiment is able to provide a framework for overcoming such pressing issues, providing analytical tools to link timescales and levels of embodiment on a case-by-case basis, uncovering the underlying developmental processes, and providing a platform to clarify and, ultimately, bridge disciplinary boundaries among the involved research fields.

The proposed framework is not intended to serve as a guideline for one comprehensive embodiment research project, but to serve as a foundation for structuring a highly interdisciplinary research field and to allow for conceptual anchor points for interdisciplinary research endeavors. Building on both environmental and agency approaches to embodiment, as well as key concepts of developmental theory, the framework motivates the question of how a specific expression of embodied experiences relates to the process of incorporation of these experiences, and vice versa, based on the underlying developmental processes. The way to reconstruct these interrelations will be specific for each embodied function, and it will have to take into account not only the rise of a function, but also its maintenance and the regulation of loss. The developmental perspective allows to, *first, connect different timescales of embodiment* based on function-specific developmental pathways. It, *second*, allows to *relate different system levels involved in embodiment processes to one another* as they develop over the lifespan, based on their physiological and functional interconnectedness. *Third*, it allows to *clarify disciplinary boundaries and their related criteria of embodiment*, which are set according to detection methods and discipline standards. The translation of different embodiment criteria between levels, e.g., the behavioral level, the level of neural activity, and the genetic and epigenetic level, heavily depends on the knowledge about the specific developmental interconnectedness between these levels and related underlying developmental pathways for each specific embodied function under study. Here in particular, further research is needed, as such translation processes also provide the basis for cross-level data integration.

First cross-disciplinary examples, as presented in section Developmental Embodiment Research: Cross-Disciplinary Examples, already point toward the productivity of such a framework, but expansion to further research disciplines is needed to fill the knowledge gaps hindering an integrative conceptualization of embodiment. For that, we propose researchers should focus on transmission hubs across two or three levels for a specific embodied function or phenomenon, aiming at identifying developmental cascades which enable cross-level effects of embodiment. These studies would depend on the knowledge about the different developmental timescales of embodied functions and sensitive periods for the incorporation of embodied experiences at the different involved levels. Our proposed framework explicitly aims at providing a matrix and platform to bridge these different developmental timescales in the study of specific embodiment phenomena and, thus, has the potential to advance cross-level, cross-disciplinary embodiment research in the short and long run.

## Author Contributions

The first draft of the manuscript was written by VL and MK, and all authors contributed to and commented on previous versions of the manuscript. All authors read and approved the final manuscript.

## Conflict of Interest

The authors declare that the research was conducted in the absence of any commercial or financial relationships that could be construed as a potential conflict of interest.

## Publisher's Note

All claims expressed in this article are solely those of the authors and do not necessarily represent those of their affiliated organizations, or those of the publisher, the editors and the reviewers. Any product that may be evaluated in this article, or claim that may be made by its manufacturer, is not guaranteed or endorsed by the publisher.
